# Beyond Training Stability: The Need for Clinical Generalizability and Explainability in Self‐Supervised ECG Models

**DOI:** 10.1111/anec.70219

**Published:** 2026-06-23

**Authors:** Cem Korucu, Bülent Görenek

**Affiliations:** ^1^ Department of Cardiology Suruç State Hospital Şanlıurfa Türkiye; ^2^ Department of Cardiology, Faculty of Medicine Eskisehir Osmangazi University Eskisehir Türkiye

**Keywords:** artificial intelligence, contrastive learning, ECG classification, electrocardiography, few‐shot learning, self‐supervised learning

To the Editor,

1

We read with considerable interest the study by Zeng et al. evaluating self‐supervised contrastive pretraining for extreme few‐shot electrocardiogram (ECG) classification. The authors should be congratulated for addressing a clinically relevant challenge in cardiovascular artificial intelligence: the scarcity of high‐quality annotated ECG datasets.

The study elegantly demonstrates that self‐supervised learning (SSL) improves training stability and substantially reduces optimization collapse under severe label constraints. These findings support previous evidence suggesting that representation learning from unlabeled ECG signals can improve downstream performance and label efficiency (Kiyasseh et al. 2021; Sarkar and Etemad 2022).

However, several considerations deserve attention before broad clinical implementation can be envisioned.

First, the proposed framework was developed and validated exclusively using the PTB‐XL database. While PTB‐XL is among the most widely used ECG datasets, model performance often declines when applied to external populations because of differences in demographics, acquisition hardware, preprocessing pipelines, and disease prevalence. External validation across multicenter datasets remains essential to establish generalizability (Strodthoff et al. 2021).

Second, the study focused on five rhythm categories. Although this design is appropriate for evaluating methodological robustness, real‐world ECG interpretation encompasses a broader spectrum of abnormalities, including myocardial ischemia, inherited arrhythmia syndromes, cardiomyopathies, conduction disturbances, and infiltrative diseases. Whether SSL confers similar benefits in these clinically complex settings remains uncertain.

Third, explainability represents a critical prerequisite for trustworthy clinical artificial intelligence. Current regulatory and reporting frameworks emphasize transparency, reproducibility, and interpretability in medical AI systems (Liu et al. 2020; Collins and Moons 2019). While SSL improves optimization stability, clinicians may remain reluctant to adopt models whose decision‐making processes cannot be adequately explained.

Finally, the principal benefit observed in the study appears to be enhanced reproducibility rather than a dramatic increase in classification accuracy. Future investigations should determine whether these methodological improvements translate into clinically meaningful gains in diagnostic accuracy, workflow efficiency, and patient outcomes.

Overall, this study represents an important step toward robust ECG‐based artificial intelligence. Future work incorporating external validation, explainable AI methodologies, and broader diagnostic tasks may further facilitate clinical translation.

## Author Contributions

Cem Korucu: Conceptualization, literature review, data interpretation, drafting of the manuscript, and critical revision of the manuscript. Bülent Görenek: Conceptualization, supervision, critical revision of the manuscript for important intellectual content, and final approval of the version to be published. Both authors contributed to the article and approved the submitted version.

## Funding

The authors have nothing to report.

## Conflicts of Interest

The authors declare no conflicts of interest.

## Data Availability

Data sharing not applicable to this article as no datasets were generated or analysed during the current study.
